# Prevalence and factors associated with energy drink consumption among Boitekanelo College students, Botswana: a cross-sectional study

**DOI:** 10.3389/fnut.2026.1887690

**Published:** 2026-08-05

**Authors:** Ellen Tsholofelo, Nankie Ramabu, Thabang Thuto Gidion, Kefentse Mosarwane, Tshiamo Mosimanegape, Mpho Miriam Marunkwane, Monnye Kgamme, Wame Itireng, Sebeletsang Mosarwa, Kago Anthony Ditshego, Atlarelang Prince Kgosi, Tshenolo Mokgole, Sarah Tshepho Pona Matenge, Oswell Khondowe, Gabalape Arnold Sejie

**Affiliations:** 1 Department of Health Promotion, School of Public Health, Boitekanelo College, Gaborone, Botswana; 2 Department of Counselling, School of Rehabilitation Sciences, Boitekanelo College, Gaborone, Botswana; 3 Department of Healthcare Service Management, School of Leadership and Management, Boitekanelo College, Gaborone, Botswana; 4 School of Rehabilitation Sciences, Boitekanelo College, Gaborone, Botswana; 5 School of Public Health, Boitekanelo College, Gaborone, Botswana

**Keywords:** associated factors, Botswana, caffeine consumption, college students, dietary habits, energy drinks

## Abstract

**Introduction:**

Over the past two decades, the global energy drink market has grown substantially, with college students emerging as a key demographic both internationally and in Botswana. Despite widespread consumption, energy drink use has been epidemiologically linked to adverse health effects. This study aimed to determine the prevalence of energy drink consumption and its associated factors among students at Boitekanelo College.

**Methods:**

An analytical cross-sectional study was conducted among students using an adapted, self-administered structured questionnaire. Participants were selected through stratified random sampling. Data were analyzed using descriptive statistics, and Pearson’s chi-square tests were performed to assess associations between energy drink consumption and socio-demographic characteristics.

**Results:**

A total of 353 participants participated in the study. The mean age was 21.61 ± 3.36 years. The prevalence of energy drink consumption was 59.0%. Among the variables examined, program of study was significantly associated with energy drink consumption (χ^2^ (14) = 36.24, *p* = 0.003). The association showed moderate effect size (Cramér’s V = 0.32), indicating moderate practical significance. No statistically significant associations were observed between energy drink consumption and age, sex, program level, year of study, marital status, or knowledge level.

**Conclusion:**

Energy drink consumption was highly prevalent among students at Boitekanelo College, indicating that energy drink use is widespread across the student population and may be driven more by contextual rather than individual-level factors. These findings underscore the need for multifaceted, evidence-informed health promotion strategies to encourage healthier beverage choices within higher education settings.

## 1 Introduction

Energy drink (ED) consumption has become a growing public health issue over the past few decades. Despite ongoing debates regarding their safety and potential benefits, EDs have been associated with adverse health outcomes, particularly cardiovascular morbidity and mortality (1). The aggressive advertising of EDs by production companies and sales agencies has made the consumption of these beverages highly appealing to young adults, students, and athletes. As a result, the global energy drink industry has experienced rapid growth, and it is predicted to generate $108 billion in profit by 2031 (2).

Energy drinks contain varying amounts of caffeine, typically ranging from 50 to 550 mg per can or bottle, along with ingredients such as taurine, l-carnitine, carbohydrates, glucuronolactone, vitamins, and herbal additives such as ginseng and guarana (3). The combined negative effects of these ingredients, particularly their high caffeine content, have been associated with several adverse health outcomes. The negative effects of ED consumption are primarily linked to the excessive sympathetic nervous system, which may result in irritability, anxiety, restlessness, insomnia, digestive issues, tremors, increased heart rate, and psychomotor agitation (4). Furthermore, the co-consumption of EDs with other alcoholic beverages, and drugs is a common practice among young people. This behavior has been associated with impaired behavioral regulation and increased engagement in risky behaviors, including unsafe sexual practices (5, 6). In addition, mixing EDs with alcohol may mask the sensation of intoxication, potentially leading to excessive alcohol consumption and increased risk-taking behaviors (7). Despite these concerns, oversight from regulatory bodies remains limited in many settings. For instance, the Food and Drug Administration (FDA) in the United States has been criticized for lacking comprehensive regulatory guidelines for EDs, while numerous low- and middle-income countries lack comprehensive regulations governing the advertising, labeling, and distribution of these‘ products (8, 9).

College students are a particularly vulnerable population regarding ED consumption. The transition to higher education is often accompanied by increased academic pressures, inconsistent sleep patterns, and shifting social lifestyles. As a result, students frequently consume EDs to improve concentration and maintain alertness during extended study periods (10). Globally, studies have reported wide variations in the prevalence of ED consumption among tertiary students. In North America, prevalence ranges between 36.4 and 70.1% (11, 12), while approximately 38% of college students in Central and South America reported similar consumption patterns (13, 14). In Asia and the Middle East, prevalence varies widely, with reported rates such as 29.3% in Riyadh (15) and 74.88% at Taif University in Saudi Arabia (16). Gender differences have also been observed, with some studies reporting higher consumption among male and female students, suggesting that sociocultural and environmental factors may influence consumption patterns (14, 16–18).

Recent evidence highlights growing concern surrounding the consumption of alcohol mixed with energy drinks (AmED) among university students (6). A systematic review and meta-analysis of 42 studies reported a worldwide prevalence of 37%, with notable geographical variation (19). Consumption of AmED was consistently associated with higher alcohol intake, risky behaviors such as drunk driving and unsafe sex, and adverse health outcomes, including psychological distress and sleep disorders (19). Although a slight decline in use has been observed, continuous monitoring and targeted educational interventions remain essential to mitigate the public health risks posed by this practice.

Several studies conducted across Africa have also reported high levels of ED consumption among university students, although prevalence and associated determinants vary across settings. A study conducted in Kano, Nigeria, reported that among the 381 respondents, 67.0% had used EDs in the previous 30 days, and 23.9% were current consumers, with Power Horse being the most popular brand (20). A cross-sectional study conducted in Mahikeng, Northwest Province, South Africa, reported a significant correlation (*p* < 0.05) between students’ use of EDs and their participation in sports (21). Across the studies, consumption habits were strongly influenced by age, sex, ethnicity, and place of residence, highlighting the need for regulatory oversight and targeted awareness programs to reduce potential health concerns.

In Botswana, the burden of non-communicable diseases, including diabetes, cardiovascular diseases and mental health conditions, has been increasing over the past decade. Evidence suggests that excessive caffeine and ED consumption may contribute to these conditions (22). Despite the growing availability and popularity of EDs in the country, there is a paucity of research examining their consumption patterns and associated risk factors among college students. This lack of context specific evidence limits the development of targeted public health interventions aimed at addressing lifestyle related risk factors. Furthermore, Botswana currently lacks specific regulatory policies governing marketing and sale of EDs, despite evidence of their potential adverse effects. Given this combination of rising consumption, documented health risks, and the absence of a local evidence base, ED use among college students in Botswana remains a critical, unaddressed gap in both the literature and in public health practice. Therefore, this study aimed to investigate factors associated with ED consumption among Boitekanelo College students. The findings are expected to inform institutional health promotion interventions, contribute to policy development and expand the existing body of knowledge on ED consumption among college students.

## 2 Materials and methods

### 2.1 Study design and setting

The study employed an analytical cross-sectional design and was conducted at Boitekanelo College, a leading healthcare training institution located in Tlokweng, Botswana.

### 2.2 Study population

The study population comprised students enrolled at Boitekanelo College, Tlokweng Campus, during the 2024–2025 academic year. According to the Registrar’s Office at the Tlokweng Campus, an estimated 3,004 students met the eligibility criteria. Eligible participants were students aged 18 years and older. Students who were pregnant, enrolled part-time, on block release, or undertaking field placement outside the college at the time of data collection were excluded. Participant eligibility was verified prior to data collection through confirmation of student registration records at Boitekanelo College.

### 2.3 Sample size and sampling strategies

The sample size was calculated using Yamane’s formula, where “n” represents the sample size, “N” the population size, and “e” the margin error $n=\frac{N}{1+N\,{\left(e\right)}^{2}}$ (23). Using this formula, (*N* = 3,004; e = 0.05), the required sample size was 353 participants.

Participants were selected using a stratified simple random sampling technique. The study population was stratified by program of study and year of enrolment to ensure adequate representation across academic groups. Within each stratum, eligible students were selected by simple random sampling using the lottery method without replacement until the required sample size was attained. Specifically, each eligible student was assigned a unique identification number, which was concealed on identical folded slips and placed in a secure container for random selection. This probability sampling approach ensured that each eligible student within a stratum had an equal and non-zero probability of selection, thereby minimizing selection bias and enhancing the representativeness and generalizability of the study findings (24).

### 2.4 Data collection and procedure

Data were collected using a self-administered structured questionnaire adapted from previously published studies (14, 25). The final version of the tool comprised 30 items organized in two main sections. Section A consisted of the socio-demographic characteristics of the participants, such as age, gender, level of program, program of study, level of study and marital status. Section B assessed participants’ knowledge and consumption patterns of energy drinks (initiation of use, types of drinks consumed, places of purchase, frequency of consumption, and reasons for use). Energy drink consumption was assessed using the following question: “Do you presently consume or use energy drinks?” Response categories were (1 = “Yes” and 2 = “No.”) Participants who reported consuming EDs were subsequently asked to identify the brands consumed (e.g., Red Bull, Monster, and Reboost), indicate their frequency of consumption (daily, 2–3 times per week, 4–6 times per week), and report the number of occasions on which they consumed EDs during the preceding 30 days (1–5, 6–10, 11–15 and 16–20 occasions). The knowledge section consisted of 11 items in which students evaluated statements using a True/False format. Scoring was based on a 1 point for a correct answer and 0 for an incorrect answer, resulting in a total score ranging from 0 to 11. To categorize participants’ knowledge levels, an adopted scoring system (modified Bloom’s cut-off points) was applied, where scores < 60% (0 to 6) were categorized as poor knowledge, 60–79% (7 to 8) as moderate knowledge and > 80% (9 to 11) as good knowledge (26, 27). Consumption frequency and patterns were assessed through closed-ended multiple-choice questions with predefined response categories. The questionnaires were distributed to participants in person at the end of scheduled classes in designated lecture rooms. Participants completed the questionnaires immediately upon receipt, with an average completion time of 10 to 15 min. Trained research assistants were present throughout the sessions to address any real-time participant queries or structural clarifications regarding the questionnaire items.

### 2.5 Study variables measured

This study considered several variables, classified as independent and dependent. The independent variables consisted of socio-demographic characteristics including age, gender, program of study, level of program, year of study, marital status and knowledge level. The dependent variable, or primary outcome was defined as energy drink consumption.

### 2.6 Pretesting

To ensure the validity and reliability of the questionnaire, the adapted English version was first reviewed by a panel of six independent experts in public health and nutritional epidemiology. The experts evaluated the questionnaire for face and content validity and assessed whether the items adequately measured the intended behavioral and cognitive constructs. Each independent reviewer provided recommendations which were all considered and consolidated, resulting in the final version of the tool. The questionnaire was translated to the local language (Setswana) to ensure comparability, data quality, accessibility and inclusivity. Prior to the main data collection, the questionnaire was pretested with 53 participants (15% of the sample) by well-trained research assistants. Insights gained from pretesting led to slight modifications to improve the tool’s validity and reliability. The recorded internal consistency coefficient for the pretesting (Cronbach’s α) was 0.857.

### 2.7 Statistical analysis

Following data collection, all questionnaires were reviewed for completeness, and the data were entered into Microsoft Excel for cleaning and coding. The cleaned data set was subsequently exported to the Statistical Package for Social Sciences (SPSS) version 29.0 for analysis. Descriptive statistics, including frequencies, percentages, means, and standard deviations, were used to summarize participants’ socio-demographic characteristics and patterns of ED consumption. The association between ED consumption and selected socio-demographic variables was assessed using Pearson’s chi square test. Statistical significance was defined as a two-sided *p*-value of < 0.05.

### 2.8 Ethical considerations

Ethical approval for this study was obtained from the Botswana Health Research and Development Division (Reference No. HPRD:6/14/1) on 22nd September 2025, prior to study commencement. Participation was voluntary, and written informed consent was obtained from all participants before data collection. Participants received a detailed information sheet outlining the study objectives, procedures, potential risks and benefits, and their right to withdraw at any time without consequence. Data were collected anonymously to ensure confidentiality and encourage honest reporting of ED consumption and related factors. All data were used exclusively for research purposes and were accessible only to the research team. Participants were also provided with the principal investigator’s contact details should they require further information or wish to raise concerns regarding the study.

## 3 Results

### 3.1 Socio-demographic characteristics of participants

A total of 353 students participated in the study. All eligible participants provided written informed consent and completed the questionnaire, resulting in a response rate of 100%. Participants were aged 18 to 46 years, with a mean ( ± SD) age of 21.61 ± 3.362 years. The median age was 21 years (Interquartile range [IQR]: 19–23 years) and the mode was 19 years. See Table 1.

**TABLE 1:** Age measures of central tendency and variability (*n* = 353).

Age measures of central tendency and variability		Frequency
Mean		21.61
Median		21.00
Mode		19
Std. Deviation		3.362
Percentiles	25	19.00
	75	23.00

The majority of participants were aged between 18–22 years (61.0%, *n* = 216), female (73.7%, *n* = 260), enrolled in degree programs (72.0%, *n* = 254), and unmarried (96.9%, *n* = 342). First-year students (39.9%, *n* = 141) constituted the largest proportion of participants. Most participants demonstrated good knowledge of the health effects of ED consumption (*n* = 156, 44.2%). See Table 2.

**TABLE 2:** Socio-demographic characteristics of participants (*n* = 353).

Variables	Sub-category	Frequency	Percentage
Age	18–22	216	61.2
	23–27	113	32
	28–32	15	4.2
	33–37	7	2
	38–42	1	0.3
	43–46	1	0.3
Gender	Female	260	73.7
	Male	91	25.8
	I prefer not to say	2	0.6
Level of program	Degree	254	72
	Diploma	60	17
	Certificate	39	11
Program of study	Community development	6	1.7
	Healthcare service management	55	15.6
	Health informatics	7	2.0
	Emergency medical care	58	16.4
	Health promotion and education	30	8.5
	Nursing	10	2.8
	Occupational therapy	10	2.8
	Occupational health and safety	52	14.7
	Public health	10	2.8
	Speech and language therapy	7	2.0
	Clinical technology	13	3.7
	Pharmacy technology	43	12.2
	Healthcare assistance	26	7.4
	Dental surgery assistance	3	0.8
	Counselling	23	6.5
Year of study	Year 1	141	39.9
	Year 2	95	26.9
	Year 3	86	24.4
	Year 4	31	8.8
Marital status	Single	342	96.9
	Married	8	2.3
	Divorced	3	0.8
Knowledge level	Poor knowledge	57	16.1
	Moderate knowledge	140	39.7
	Good knowledge	156	44.2

### 3.2 Prevalence of energy drink consumption

**FIGURE 1:**
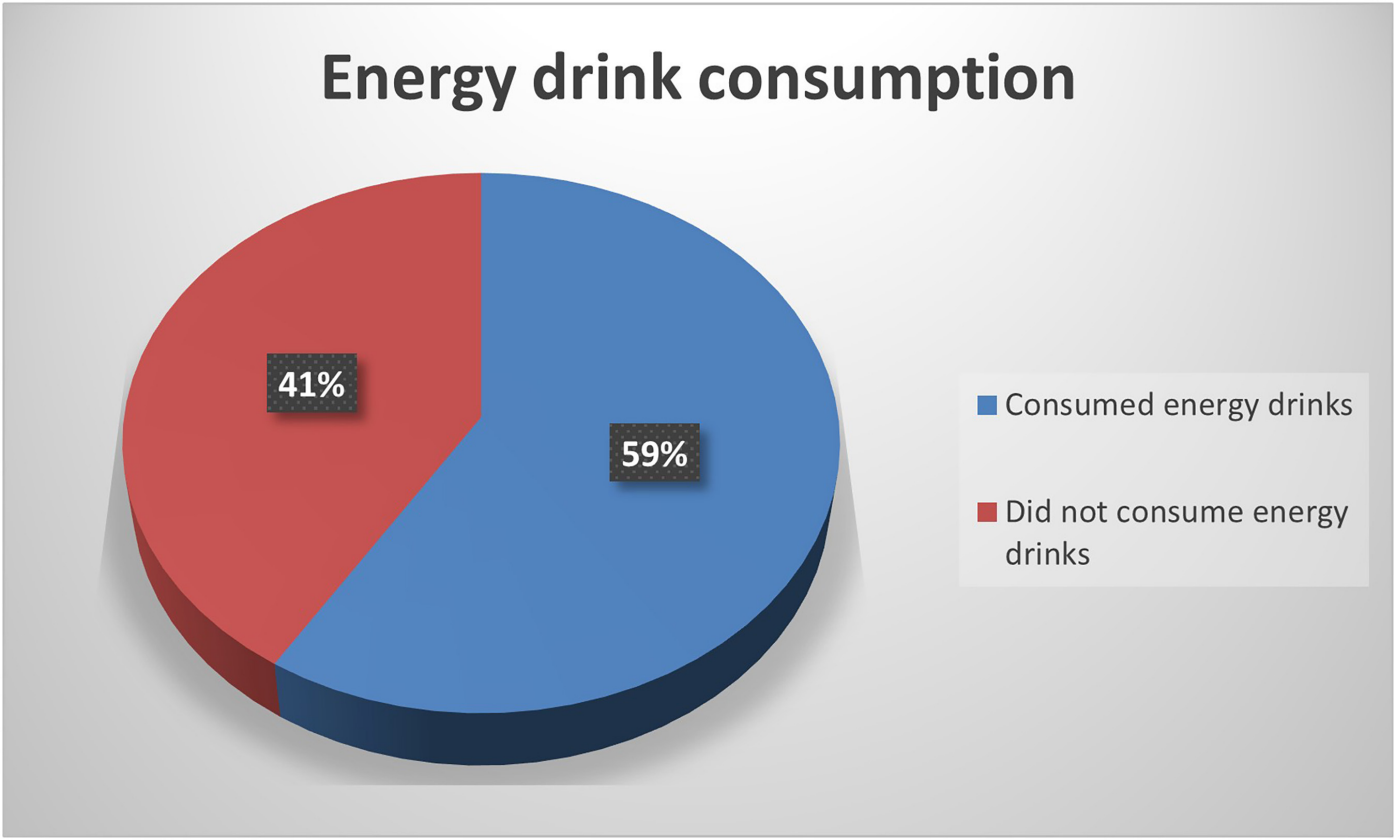
Prevalence of energy drink consumption.

Overall, more than half (59%, *n* = 208) of the participants reported consuming EDs, whereas 41% (*n* = 145) reported no consumption. See Figure 1.

### 3.3 Factors associated with energy drink consumption

Among all the variables examined, only program of study was significantly associated with ED consumption [x^2^ (14) = 36.24, *p* = 0.003]. The association demonstrated a moderate effect size (Cramer’s V = 0.32), indicating moderate practical significance. No statistically significant associations were observed between ED consumption and age, gender, level of program, year of study, marital status and knowledge level (*p* ≥ 0.05). See Table 3.

**TABLE 3:** Outcome crosstabulation (*n* = 353).

Energy drink consumption					
Variable	Sub-category	Frequency	Percentage (%)	*p*-value	Cramér’s V
Age category	18–22	127	61.1	0.385	–
	23–27	72	34.6		
	28–32	5	2.4		
	33–37	3	1.4		
	38–42	0	0		
	43–46	1	0.5		
Gender	Female	150	72.1	0.633	–
	Male	58	27.9		
	I prefer not to say	0	0.0		
Level of program	Degree	156	75	0.315	–
	Diploma	29	13.9		
	Certificate	23	11.1		
Program of study	Community development	5	2.4	0.003	0.32
	Healthcare service management	37	17.8		
	Health informatics	4	1.9		
	Emergency medical care	38	18.3		
	Health promotion and education	18	8.7		
	Nursing	7	3.4		
	Occupational therapy	3	1.4		
	Occupational health and safety	38	18.3		
	Public health	3	1.4		
	Speech and language therapy	2	1		
	Clinical technology	3	1.4		
	Pharmacy technology	29	13.9		
	Healthcare assistance	11	5.3		
	Dental surgery assistance	0	0		
	Counselling	10	4.8		
Year of study	Year 1	77	37	0.199	–
	Year 2	56	26.9		
	Year 3	57	27.4		
	Year 4	18	8.7		
Marital status	Single	202	97.1	0.909	–
	Married	4	1.9		
	Divorced	2	1		
Knowledge level	Poor knowledge	32	15.4	0.476	–
	Moderate knowledge	88	42.3		
	Good knowledge	88	42.3		

## 4 Discussion

### 4.1 Prevalence of energy drink consumption

This study investigated the prevalence of ED consumption and its association with selected socio-demographic characteristics among students at Boitekanelo College. The findings revealed a high prevalence of ED consumption among Boitekanelo College students, with more than half of participants reporting current use. This highlights the widespread consumption of EDs among college students and reinforces concerns regarding their increasing use among young adults, given the potential health risks associated with excessive consumption. This pattern is consistent with cross-sectional studies conducted among university students in Cairo and India, which similarly reported high rates of ED consumption (17, 28). These authors attributed the high prevalence to factors including academic pressure, sleep deprivation, and the widespread availability of EDs. In contrast, cross-sectional studies conducted in Italy, Puerto and Zambia reported lower prevalence rates of ED consumption (29–31); the Italian authors attributed their comparatively low rate to protective traditional dietary practices. Taken together, these observed differences across studies suggest that ED consumption is influenced not only by individual behaviors but also by broader contextual and environmental factors, including cultural norms, product availability, and branding strategies (32). The high prevalence of ED use observed in this study is a public health concern due to the documented negative effects linked to excessive consumption, which include headache, difficulty sleeping, anxiety, restlessness and cardiovascular complications (33). Although the present study did not directly assess these health outcomes, the findings underscore the need for targeted educational interventions to enhance students’ understanding of the potential health risks linked to ED consumption.

### 4.2 Factors associated with energy drink consumption

Among the socio-demographic characteristics examined, program of study was the only variable significantly associated with ED consumption [x^2^ (14) = 36.24, *p* = 0.003, Cramer’s V = 0.32]. Students in Emergency Medical Care, Occupational Health and Safety, and Healthcare Service Management reported the highest consumption. A comparable pattern was reported among Turkish students, with consumption differing significantly across faculties of medicine, sports and the arts.; the study’s authors linked this to differing academic and physical demands across fields as well as marketing targeted at physically active students (34). We interpret this association as reflecting program-specific structural demands, such as irregular clinical placement hours, shift-based rotations, and physically demanding practicums, rather than any inherent difference in the students themselves across programs. Conversely, other cross-sectional studies have reported no significant association between program of study and ED consumption (35, 36), which may reflect institutional differences in curriculum structure, clinical exposure, or placement scheduling that are absent in more classroom-based programs. Because program of study was the sole significant correlate identified in our analysis, and given its moderate effect size, this finding has the greatest practical implication for targeted intervention: health promotion efforts at Boitekanelo College would likely have greater impact if concentrated on programs with demanding clinical placement schedules rather than applied uniformly across the student body.

Although higher proportions of ED consumption were observed among students aged 18–22 years, females, degree students, first-year students and single participants, none of these socio-demographic characteristics demonstrated a statistically significant association with ED consumption. The absence of a significant association between age and ED consumption is consistent with other cross-sectional studies among university students (37), which similarly concluded that age was not an independent predictor of consumption, suggesting that ED use may be a behavior shared broadly across the traditional university-age range rather than concentrated in a specific age band. In contrast, a study among students in Saudi Arabia reported statistically significant age-related differences in consumption (38). A plausible explanation for this divergence is that Boitekanelo College’s student population is comparatively age-homogeneous (61.1% aged 18–22 years), which limits the statistical power available to detect an age effect even if a true difference existed, whereas samples with wider age dispersion may be better positioned to reveal such associations. Taken together, these findings suggest that in relatively age-homogenous student populations such as ours, ED consumption is more likely driven by shared academic and social pressures common among university life than by age-specific vulnerability. Similarly, although female students reported a higher prevalence of ED consumption than their male counterparts, gender was not significantly associated with consumption. This finding contrasts with studies that identified male sex as a significant predictor of ED use in males (31, 39), while supporting evidence from other settings where female students reported equal or higher consumption (29, 38). Differences in study context may help explain this divergence, unlike settings where ED marketing and use is culturally associated with male-dominated sporting or social contexts, Boitekanelo College’s student body is predominantly female and concentrated in health programs. This demographic composition effect offers a more parsimonious explanation for the higher female prevalence observed here than any gender-specific behavioral difference, and is consistent with program of study, rather than gender, emerging as the only statistically significant correlate of ED consumption in this study. Although students enrolled in degree programs and those in their first-year of study reported relatively higher proportions of ED consumption, neither level of program nor year of study showed statistically significant associations. This null finding is consistent with other studies that likewise found no meaningful differences in ED consumption across levels of programs and year of study (40, 41), suggesting that the academic tier of a qualification may matter less to consumption behavior than the day-to-day demands experienced within it. In contrast, other studies reported higher consumption specifically among second-year students (16, 31, 38), suggesting that the timing of peak academic pressure is program and curriculum-specific rather than governed by a universal pattern across years of study. In the current study, the absence of a significant factor suggests that ED consumption at Boitekanelo College is not confined to any single academic milestone but is instead sustained across the student journey, reflecting continuous rather than year-specific academic and social pressures. Likewise, marital status was not a statistically significant predictor. This null result is consistent with multiple cross-sectional studies conducted in Saudi Arabia, North America, and Palestine, which similarly found no significant association between marital status and ED consumption (42–45). The consistency of this null finding across such geographically and culturally diverse settings suggests that marital status appears to have limited explanatory value in this literature, plausibly because college students, regardless of setting, tend to share comparable academic obligations and lifestyle. Given both consistency of this finding across studies and limited variability of marital status within our own sample, we would caution against over-interpreting this variable in future studies of similar student populations and instead recommend that intervention design prioritize the behavioral, environmental, and academic factors already identified as more informative in this and prior work.

No statistically significant association was observed between knowledge level and ED consumption in this study, despite majority of students demonstrating moderate to good knowledge. A comparable pattern was reported in Jordan, where students demonstrated moderate overall knowledge of EDs (mean score 7.1 of 12) yet 66.0% reported having consumed them, prompting the study’s author to call for structured awareness campaigns precisely because knowledge alone appeared insufficient to influence behavior (46). A similar disconnect between awareness and behavior was reported among students in Ghana (47). Conversely, other studies conducted in Jordan and Iraq reported generally poor knowledge of ED effects among their samples (48, 49); although this represents a different baseline, it does not contradict the broader pattern, since across settings with both good and poor baseline knowledge, knowledge level has not reliably predicted consumption behavior. It is important to note, however, that because this study is cross-sectional, we cannot determine whether students’ knowledge preceded or resulted from their consumption experience, and this non-significant association should therefore be interpreted with caution rather than as evidence that health education is ineffective. Collectively, these findings suggest that knowledge-based education alone is unlikely to be a sufficient intervention on its own, and should be paired with behavioral, social and environmental approaches that address the broader context in which consumption decisions are made.

### 4.3 Limitations

This study has several limitations. Its cross-sectional design, with data collected at a single point in time, precludes causal inference between socio-demographic factors and ED consumption; in particular, it was not possible to determine whether participants’ knowledge of the health effects of EDs preceded or resulted from their consumption behaviors. Energy drink consumption was assessed through self-reported data, which may be subject to recall bias and social desirability bias. In addition, the questionnaire assessed current ED consumption without specifying a clearly defined recall period (for example, the preceding 30 days or past 12 months), which limits direct comparison with studies reporting past-month or past-year prevalence. Furthermore, the study was conducted exclusively among Boitekanelo College students in Gaborone, therefore, the findings may not generalize to other tertiary institutions in Botswana or the wider young adult population, given differences in student demographics, academic environments, and lifestyle factors elsewhere. Despite these limitations, methodological steps were taken to strengthen the study, including pretesting of the tool, sampling students across all programs offered in the college and ensuring an adequate sample size.

## 5 Conclusion

This study demonstrates that ED consumption is highly prevalent among Boitekanelo College students, highlighting it as an important public health concern within institutions of higher learning. Program of study was the only socio-demographic factor significantly associated with ED consumption, suggesting that program-specific academic contexts may influence consumption behaviors. In contrast, age, gender, level of program, year of study, marital status and knowledge of ED-related health effects were not significantly associated with consumption, indicating that ED use is widespread across the student population. Collectively, these findings further suggest that knowledge of the potential health risks alone may be insufficient to modify consumption behaviors. Consequently, comprehensive, evidence-informed interventions that integrate behavioral, educational, and environmental strategies are needed to promote healthier beverage choices among students. Future research should investigate the contextual and psychological determinants of ED consumption to inform the development of targeted and effective interventions.

### 5.1 Recommendations

Based on the findings of this study’s findings, institutions of higher learning should implement targeted health promotion interventions to raise awareness of the potential adverse health risks associated with excessive ED consumption. Institutions should also strengthen student wellness initiatives that promote healthy strategies for coping with academic stress and fatigue as alternatives to ED use. In addition, environmental and policy-based interventions, such as regulating the marketing and availability of EDs within educational settings, may complement individual strategies. Although these factors were not directly assessed in the present study, evidence from the broader literature suggests that they may help reduce excessive ED consumption among young adults. Future multi-institutional longitudinal studies are warranted to investigate the determinants, consumption patterns, and long-term health consequences of ED use among college students in Botswana, as well as to evaluate the effectiveness of intervention strategies.

## Data Availability

The raw data supporting the conclusions of this article will be made available by the authors, without undue reservation.
